# A sequential trial effect based on the motor interference effect from dangerous objects: An ERP study

**DOI:** 10.1002/brb3.1112

**Published:** 2018-09-03

**Authors:** Peng Liu, Xiaoyi Wang, Gai Cao, Jia Li, Jing Zhang, Rong Cao

**Affiliations:** ^1^ School of Public Management Northwest University Xi'an China; ^2^ School of Economics and Management Northwest University Xi'an China; ^3^ Mental Health Education Center of Northwest University Xi'an China

**Keywords:** avoidance motivation, dangerous objects, motor interference effect, motor priming paradigm, sequential trial effect

## Abstract

**Introduction:**

This study aims to investigate whether processing a prepared response toward a dangerous object in a previous trial influences subsequent trial processing.

**Methods:**

The design manipulated the Go/NoGo factor of the current trial, the target dangerousness of the previous trial and that of the current trial.

**Results:**

In current Go trials, the behavioral results revealed a classical motor interference effect in trials that were preceded by a safe trial (a longer reaction time (RT) and a larger error rate for the previous safe and current dangerous (sD) condition than for the previous safe and current safe (sS) condition). However, the motor interference effect diminished in trials that were preceded by a dangerous trial (insignificant differences in the mean RTs and error rates between the previous dangerous and current dangerous (dD) condition and the previous dangerous and current safe (dS) condition). The event‐related potential (ERP) results identified more positive P2 and parietal P3 amplitudes (indicating attentional resource allocation) for the dD condition than for the dS condition. However, the P2 and parietal P3 amplitudes of the sD condition did not significantly differ from those of the sS condition.

**Discussions:**

These results support the hypothesis that the avoidance motivation elicited by a dangerous target in a previous trial may indicate a dangerous situation, which leads to recruitment of more attentional resources allocated to the subsequent dangerous trial. Therefore, RTs are improved and errors are reduced in the consecutive dangerous condition, subsequently decreasing the motor interference effect in trials preceded by a dangerous trial compared with trials preceded by a safe trial. However, analysis of current NoGo trials revealed that none of the main effects or interactions reached significance in both the behavioral and ERP results, indicating that the hypothesis holds true only if the prepared response needs to be executed.

## 1 INTRODUCTION

How to effectively avoid accidents that occur during the manipulation of machines (especially dangerous elements in machines) is becoming increasingly important in safety management. To reduce the occurrence of accidents, the mechanisms for processing a prepared response when facing a dangerous object (a dangerous object is defined as an object that poses a potential threat to humans) must be investigated. Evidence shows that observing a picture of a dangerous object can delay a prepared response toward it (Anelli, Borghi, & Nicoletti, 2012); this phenomenon is called the motor interference effect from a dangerous object. Existing research has investigated the origin of this effect from both behavioral (Anelli et al., 2012) and event‐related potential (ERP) perspectives (Liu, Cao, Chen, & Wang, 2017). Moreover, in a working environment filled with dangerous elements, whether processing a prepared response when facing a dangerous object in a previous trial influences subsequent trial processing (i.e., a sequential trial effect based on the motor interference effect from dangerous objects) should also be investigated. Clarifying this issue may yield experimental evidence regarding how successively presented dangerous objects are processed in a work environment. To our knowledge, although studies have addressed the origin of the motor interference effect from dangerous objects, little is known regarding the sequential trial effect based on the motor interference effect from dangerous objects. This study aimed to investigate this issue by further analyzing data from Liu et al. (2017).

In cognitive psychology, the sequential trial effect has been investigated with an emphasis on response interference tasks, such as the Flanker task (Ullsperger, Bylsma, & Botvinick, 2005), the Stroop task (Larson, Kaufman, & Perlstein, 2009), and the Simon task (Kerns, 2006; Notebaert & Verguts, 2011). In these tasks, task‐relevant and task‐irrelevant stimuli features can be either congruent or incongruent and, thereby, create nonconflict or conflict trials, respectively. Reaction times (RTs) and error rates are increased in conflict versus nonconflict trials. For example, in the Flanker task, participants are required to give a left‐ or right‐hand response to the direction of a central target arrow while ignoring congruent (e.g., < < < < <) or incongruent (e.g., < < > < <) flanker arrows. Typical results reveal a conflict effect indicated by delayed RTs and increased error rates in incongruent versus congruent trials. Interestingly, this conflict effect is modulated by the congruency of previous trials. Specifically, the conflict effect is usually reduced in trials that are preceded by incongruent trials (a previous incongruent and current incongruent (iI) condition minus a previous incongruent and current congruent (iC) condition) compared with trials that are preceded by congruent trials (a previous congruent and current incongruent (cI) condition minus a previous congruent and current congruent (cC) condition). This phenomenon is called the sequential trial effect or the Gratton effect (Duthoo, Abrahamse, Braem, Boehler, & Notebaert, 2014; Egner, 2007; Gratton, Coles, & Donchin, 1992). An explanation of the sequential trial effect can be found in the conflict‐monitoring account, that is, once the cognitive system detects a response conflict in an incongruent trial, an increase in cognitive control is observed in the following trial, which subsequently leads to reduced interference if the subsequent trial is also incongruent (Botvinick, Braver, Barch, Carter, & Cohen, 2001; Botvinick, Cohen, & Carter, 2004; Carter et al., 1998).

This sequential trial effect is modulated by avoidance motivation (Hengstler, Holland, Van Steenbergen, & Van Knippenberg, 2014). Avoidance motivation is defined as motivation that keeps one out of danger. Hengstler et al. (2014) manipulated avoidance and approach motivations (approach motivation can help one attains essential outcomes including food, drinks, etc.) by instructing participants to maintain an approach or avoidance motor action with one hand while the Flanker task is performed with the other hand. The results of the Flanker task revealed an increase in the sequential trial effect (cI – cC) – (iI – iC) for the avoidance motivation task compared with the approach motivation task. This result may have occurred because avoidance motivation indicates potentially dangerous situations, and thus, the current situation was more carefully examined, which led to enhanced cognitive control and, in turn, recruited more attentional resources in response to consecutive conflicts. Therefore, the conflict effect was reduced in the consecutive conflict (iI) condition compared with the cI condition, thus increasing the sequential trial effect in the avoidance motivation task compared with the approach motivation task.

This study aims to extend this hypothesis by exploring trial‐by‐trial influence to determine whether processing a prepared response toward a dangerous object in a previous trial influences subsequent trial processing. Evidence indicates that observing a picture of a dangerous object can elicit avoidance motivation, which in turn delays RTs in response to dangerous objects (Anelli et al., 2012). According to the hypothesis suggested by Hengstler et al. (2014), the avoidance motivation elicited by a dangerous target may reduce the conflict effect if the subsequent trial is also dangerous (i.e., the motor interference effect may be reduced in the consecutive dangerous condition). However, this trial‐by‐trial influence of dangerous objects on the motor interference effect may differ from the influence of a maintained avoidance motor action on the Flanker task, as in Hengstler et al. (2014). First, the trial‐by‐trial influence was characterized by phase (short‐term) avoidance motivation elicited by a previous trial, which differs from state (long‐term) avoidance motivation maintained across a block in Hengstler et al. (2014). Second, unlike the approach movement toward an object, avoidance motivation elicited by a dangerous object is more habitually (involuntarily) processed because dangerous objects naturally induce an avoidance effect in humans (Anelli, Ranzini, Nicoletti, & Borghi, 2013; Coello, Bourgeois, & Iachini, 2012; Cole, Balcetis, & Dunning, 2013). However, maintaining an avoidance motor action versus an approach motor action, as in Hengstler et al. (2014), may elicit consciously controlled processes. Evidence suggests that involuntary and voluntary processes have substantially different neural bases (Eimer & Schlaghecken, 2003). Therefore, the trial‐by‐trial influence of dangerous objects on the motor interference effect must be individually investigated via a more suitable paradigm.

Accordingly, this study adopted a motor priming paradigm mixed with a Go/NoGo task to investigate the trial‐by‐trial influence of dangerous objects on the motor interference effect. This paradigm was adopted by Liu et al. (2017) to imitate a motor situation involving executing or withholding a prepared motor reaction in the context of an emergent dangerous object. Pictures of a left or right hand were used as primes, and green (Go signal) or red (NoGo signal) circles superimposed on dangerous or safe objects were used as targets. The participants were instructed to prepare for the corresponding key press with the hand that was consistent with the handedness of the prime and to not to execute the key press until a Go signal appeared. The design manipulated the dangerousness of the target objects (safe vs. dangerous) and the Go/NoGo (Go vs. NoGo) signal superimposed on the targets. The results revealed delayed RTs and increased errors together with larger parietal P3 amplitudes (i.e., the P3b component, which reflects attentional allocation processing (Isreal, Chesney, Wickens, & Donchin, 1980)) in the dangerous condition compared with the safe condition in the Go trials. However, these behavioral and ERP differences did not emerge in the NoGo trials. The results indicated that the motor interference effect from dangerous objects may originate from increased attentional resource allocation for dangerous objects. Furthermore, this effect emerged only if the prepared response was executed (i.e., Go trials) because execution of the prepared response imitated an approach movement toward the dangerous targets, which may pose a threat to subjects.

Based on these findings, this study manipulated not only the dangerousness of the target objects and the Go/NoGo factor of the current trial but also the dangerousness of the target objects of the previous trial (previous safe vs. previous dangerous). According to Liu et al. (2017), the responses to the dangerous targets were affected by the attentional resources allocated to the targets in the current Go trials. This study further investigated whether the attentional resources assigned to the dangerous target in the current trial would be affected by the avoidance motivation elicited by the dangerous target in the previous trial. According to Hengstler et al. (2014), the avoidance motivation elicited by a dangerous target in a preceding trial may indicate a dangerous situation, and more attentional resources may be prepared to evaluate the target in the subsequent trial. If the subsequent trial contains dangerous elements, deeper processing of the dangerous target can recruit more attentional resources and, thereby, facilitate the response to the dangerous trials. However, the prepared attentional resources may not be required for the current safe trial because rare dangerous elements exist. This result leads to a reduced motor interference effect in trials that are preceded by a dangerous trial (a previous dangerous and current dangerous condition (dD) minus a previous dangerous and current safe condition (dS)) compared with trials that are preceded by a safe trial (a previous safe and current dangerous condition (sD) minus a previous safe and current safe condition (sS)). Because a safe target in a previous trial indicates that nothing important has occurred in the environment, a classical motor interference effect is expected. Furthermore, the above hypothesis may only take effect in current Go trials because the motor interference effect emerged only if the prepared response was executed (Liu et al., 2017). Together, in current Go trials, an interaction is expected between the dangerousness of the current trial and the dangerousness of the previous trial in both behavioral and ERP results. We expected a reduction in the difference in RTs and error rates in the dD minus dS condition compared with in the sD minus sS condition because increased attentional resources are allocated to the consecutive dangerous (dD) condition, which may accelerate RTs and reduce errors. Moreover, the difference in parietal P3 amplitudes, which reflect the allocation of attentional resources (Isreal et al., 1980), should be larger in the dD minus dS condition than in the sD minus sS condition. Additionally, we predicted larger P2 amplitudes in the dD minus dS condition than in the sD minus sS condition. Previous studies have indicated that the P2 component is associated with the detection of threats and that deeper feature detection will increase the P2 amplitudes accordingly (Correll, Urland, & Ito, 2006). Therefore, if the avoidance motivation elicited by a dangerous target in a previous trial indicates a dangerous situation, which leads to deeper processing of the dangerous target in the current trial, a more positive P2 amplitude should emerge in the dD condition than in the dS condition. In contrast, the P2 amplitude difference should decrease between the sS and sD conditions. However, Liu et al. (2017) reported that attentional resources allocated to the targets were not influenced by target dangerousness in current NoGo trials. Therefore, we speculated that an insignificant effect as a function of the dangerousness of the current trial and previous trial would be observed in both behavioral and ERP results.

## 2 METHODS

### 2.1 Participants

Twenty right‐handed undergraduates (aged between 18 and 23 years, mean age = 20 years, 9 males and 11 females) participated in the experiment. All the participants had normal or corrected‐to‐normal visual acuity and were not color blind. The experiment was performed in compliance with the relevant institutional guidelines and was approved by the Ethics Committee of the School of Public Management of Northwest University. The participants provided written informed consent and were compensated with 50 yuan RMB.

### 2.2 Materials and apparatus

**Figure 1:**
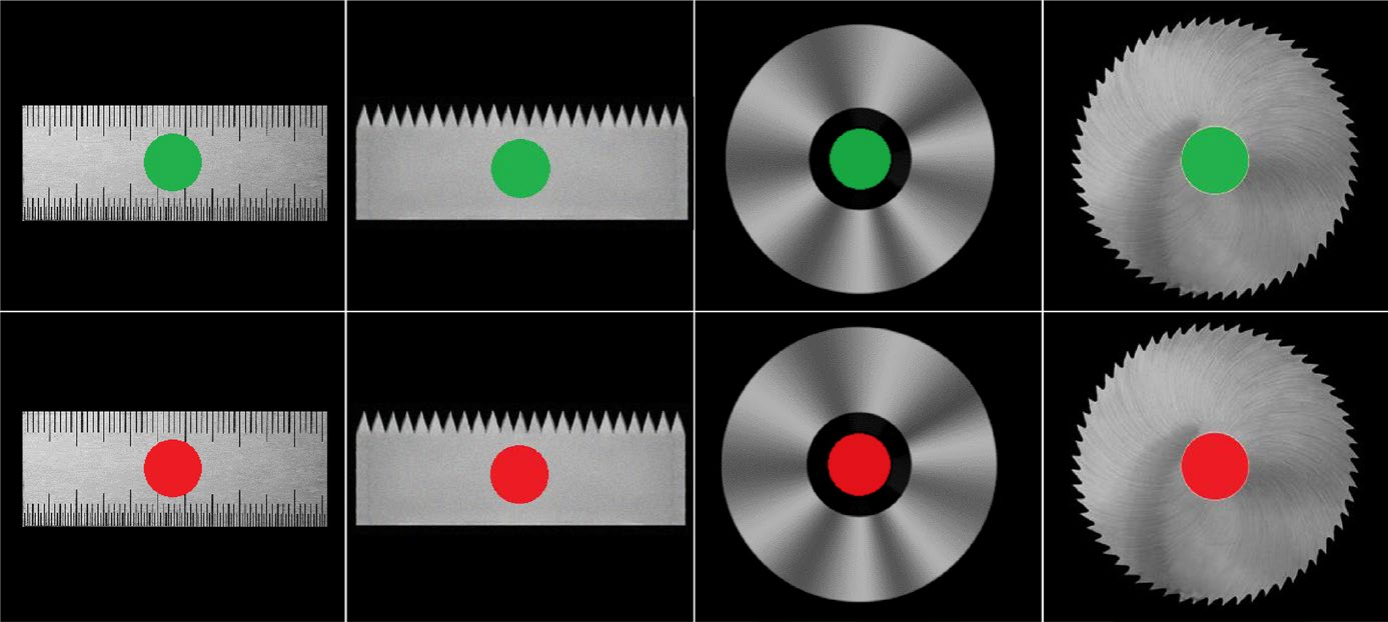
Schematic representation of the target stimuli

The experimental stimuli were identical to those in our previous study (Liu et al., 2017). The primes included pictures of a left or right hand with a partial forearm (13° horizontally and 11° vertically), and the targets included pictures of a green or red circle (2° horizontally and 2° vertically) superimposed on a safe or dangerous object (Figure 1). The safe objects included a ruler (10° horizontally and 3.8° vertically) and a disk (8.8° horizontally and 8.8° vertically); the dangerous objects included a shape‐matched rectangular saw blade (10° horizontally and 3.8° vertically) and a round saw blade (8.8° horizontally and 8.8° vertically). The assessments of target dangerousness indicated that the rectangular saw blade and the round saw blade were more dangerous than the ruler and the disk (see footnote 2 in Liu et al. (2017)). The targets were presented in the center of the screen, and the left‐ or right‐hand primes were presented 2° to the left or right of the fixation point to imitate a spatially matched grasping situation. All the stimuli were presented on a black background. The stimuli presentations were controlled with E‐Prime software (version 2.0, Psychology Software Tools, Inc., Pittsburgh, PA, USA, http://scicrunch.org/resolver/SCR_009567), which was run on a standard PC linked to a 17‐in CRT monitor (60‐Hz refresh rate). A NeuroScan system (NeuroScan Inc., Herndon, VA, USA, http://scicrunch.org/resolver/SCR_015818) was used to record the electroencephalogram (EEG) data.

### 2.3 Procedure

**Figure 2:**
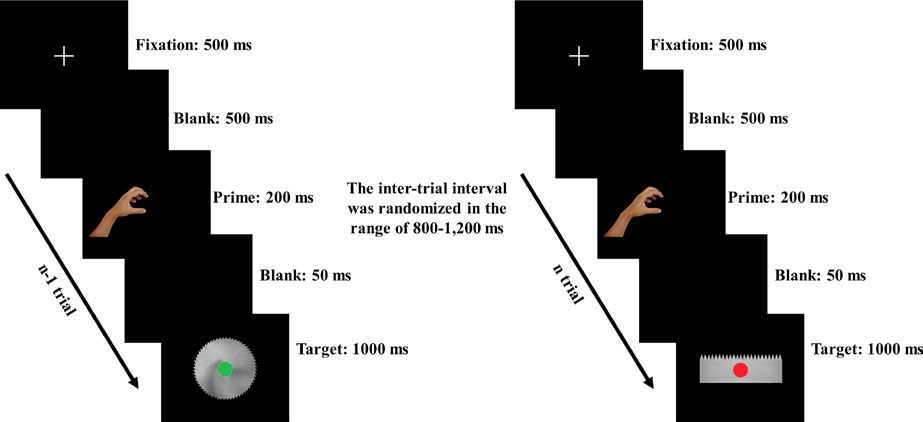
Schematic representation of the trial procedure

The participants were seated in a dimly lit chamber with a computer screen placed 60 cm in front of their eyes. The center of the screen was in the center of the participant's horizontal sightline straight ahead at a fixed level. Each trial was initiated with a central fixation cross (500 ms) followed by a blank screen (500 ms). A left‐ or right‐hand prime was subsequently presented for 200 ms, followed by a blank screen for 50 ms, and a final target (1,000 ms) was successively presented. Note that the target display was terminated if the response was executed within 1,000 ms. The intertrial interval was randomized in the range 800–1,200 ms (Figure 2). The participants were instructed to maintain their central eye fixation and to respond according to the Go/NoGo signal superimposed on the target. Specifically, they were instructed to prepare a left‐ or right‐hand response that corresponded to the handedness of the prime and to refrain from executing the response until a Go signal (a green circle) appeared. When a Go signal appeared, the participants were asked to execute the prepared response as fast as possible. The participants were instructed to execute a left‐hand response by pressing the “F” key using the index finger of their left hand and to execute a right‐hand response by pressing the “J” key using the index finger of their right hand on an English keyboard. Alternatively, when a NoGo signal (a red circle) appeared, the participants were asked to withhold the prepared response.

The experimental design manipulated the dangerousness of the target objects in the previous trial (i.e., the n‐1 trial, previous safe vs. previous dangerous), the dangerousness of the target objects in the current trial (i.e., the n trial, current safe vs. current dangerous) and the Go/NoGo signal of the current trial (current Go vs. current NoGo). Note that the Go/NoGo signal of the previous trial was not manipulated in the design because the avoidance motivation is inevitably elicited by the dangerous object in the previous dangerous trial regardless of whether the trial was Go or NoGo. The trials were pseudorandomly sequenced to yield equal proportions of previous safe and current safe (sS), previous safe and current dangerous (sD), previous dangerous and current safe (dS), and previous dangerous and current dangerous (dD) conditions. The two levels of the current Go/NoGo factor were counterbalanced in each of the four (sS, sD, dS, and dD) conditions in equal proportions. To eliminate a perceptual repetition effect, the same object did not appear in adjacent trials. Three blocks with 129 trials were assigned in each block for a total of 387 trials. Each block began with a safe trial, and this first trial was excluded from the data analysis. In total, the experiment consisted of 384 effective trials, with each sS, sD, dS, and dD condition comprising 48 repetitions each in the Go and NoGo conditions. A 16‐trial practice phase was conducted prior to the actual experiment, and the formal experiment did not begin unless the participant's percentage of correct responses in the practice phase exceeded 87.5%. The participants were allowed a two‐minute break after each block and were encouraged to take longer breaks when necessary.

### 2.4 EEG recording and processing

The EEG data were continuously recorded (bandpass filter 0.05–100 Hz, sampling rate 250 Hz) with a Neuroscan Synamp 2 Amplifier using an electrode cap with 64 Ag/AgCl electrodes mounted according to the extended international 10–20 system and referenced to the tip of the nose. The vertical and horizontal electrooculograms (i.e., the VEOG and HEOG) were recorded with two pairs of electrodes that included one pair placed above and below the left eye and another pair placed 10 mm from the lateral canthi. The electrode impedance was maintained below 5 kΩ throughout the experiment.

The EEG data were preprocessed using EEGLAB (Version 13, http://scicrunch.org/resolver/SCR_007292) (Delorme & Makeig, 2004). Continuous EEG data were high‐pass filtered at 1 Hz, low‐pass filtered at 30 Hz and re‐referenced to the bilateral mastoid electrodes. The EEG data were subsequently segmented and time‐locked to the target onset in epochs of 3,000 ms with a presplicing point of 1,000 ms. The epoched data were corrected to baseline using the 1,000 ms prior to the target onset. Epochs with large artifacts (i.e., those that exceeded ± 100 μV) and incorrect responses were removed, and the few channels with poor signal quality were interpolated using the EEGLAB toolbox. The trials that were contaminated by eye blinks (i.e., when the scalp topographies suggested activities near the eyes and the power was concentrated at low frequencies) and movements (i.e., scalp topographies that were oriented toward the left or right with positive values on one side and negative values on the other and a power concentrated at low frequencies) were corrected using an independent component analysis (ICA) algorithm (Delorme & Makeig, 2004). Consequently, the preprocessing step rejected an average of 12% of the epochs as contaminated across all the participants and conditions. The numbers of artifact‐free trials obtained for the sS, sD, dS, and dD conditions in current Go trials were 41.9 ± 3.5, 41.4 ± 4.1, 41.8 ± 3.9, and 41.9 ± 5.0, respectively; the numbers of artifact‐free trials obtained for the sS, sD, dS, and dD conditions in current NoGo trials were 41.7 ± 4.7, 42.6 ± 4.4, 42.5 ± 5.2, and 42.9 ± 4.2, respectively. Statistical analyses revealed insignificant differences in the effective trial numbers between these conditions in the Go trials (*p*‐values = 0.77) and NoGo trials (*p*‐values = 0.35). The artifact‐free data were resegmented, initiated from the target onset to 600 ms afterward, and referenced to baseline −200 to 0 ms prior to the target onset. The extracted average waveforms for each participant and condition were used to calculate the grand‐average waveforms.

### 2.5 Statistical analysis

To accommodate the hypothesis, two‐way repeated‐measures ANOVAs were used to analyze the mean RTs and error rates separately in the Go and NoGo trials. The independent variables included the dangerousness of the previous trials and the dangerousness of the current trials. Note that the incorrect response trials and the NoGo trials were excluded from the RT analysis.

**Figure 3:**
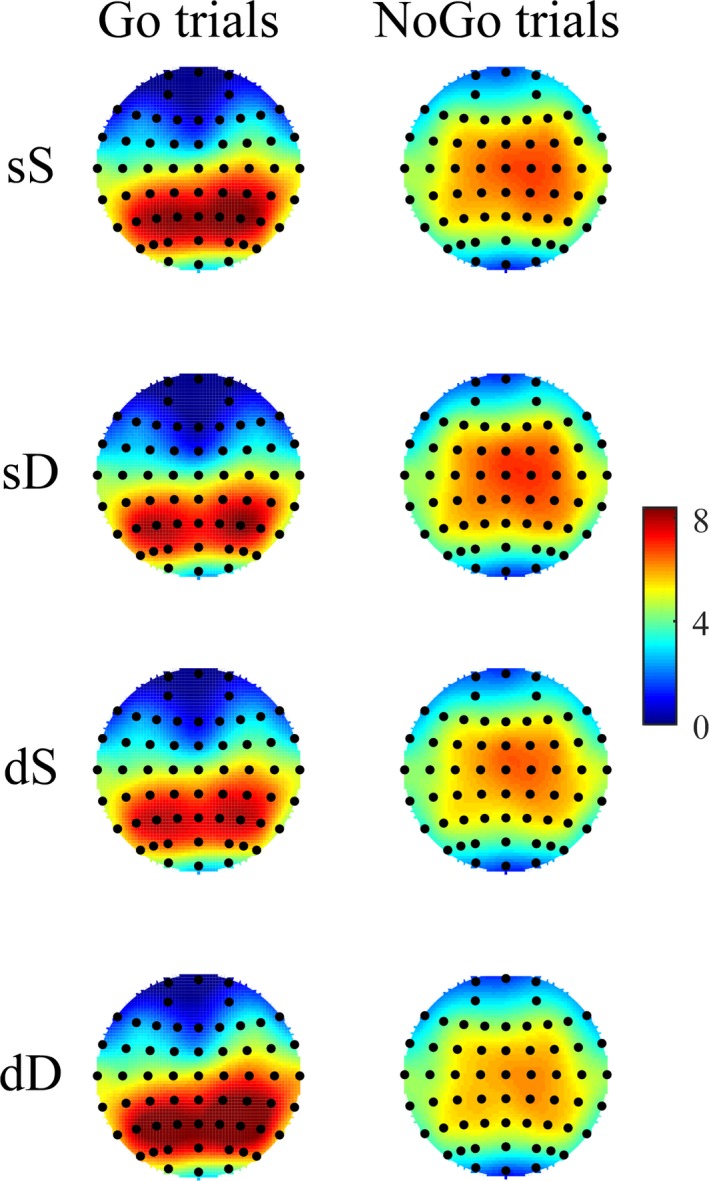
Grand‐average topographic plots of the parietal P3 component. Left panel: Scalp topographies in the 300‐ to 420‐ms time windows in current Go trials as a function of the dangerousness of the previous trial and the current trial. Right panel: Scalp topographies in the 300‐ to 460‐ms time windows in current NoGo trials as a function of the dangerousness of the previous trial and the current trial

The ERP data were also separately analyzed in the Go and NoGo trials. The parietal P3 component is usually analyzed via selection of the Pz electrode in the mid‐parietal area. However, the topographical maps in the 300‐ to 420‐ms time window in the Go trials and those in the 300‐ to 460‐ms time window in the NoGo trials exhibited right‐side lateralization in this study, as in Liu et al. (2017) (Figure 3). To utilize more channel signals, three parietal scalp regions of interest (SROIs) corresponding to the left (the average of the CP3, CP5, P3, and P5 electrodes), middle (the average of the CPz, P1, Pz, and P2 electrodes) and right (the average of the CP4, CP6, P4, and P6 electrodes) parietal areas were selected for analysis of the parietal P3 amplitudes. Furthermore, the P2 component was analyzed at the mid‐frontal SROI according to Liu et al. (2017), which was calculated as the average of the F1, Fz, F2, and FCz electrodes. Note that the inspection of the grand waveforms indicated differences between conditions in the N2 and frontal P3 components. Although the hypothesis was not associated with these components, they were also analyzed to investigate whether unanticipated differences existed among conditions.

Based on these SROIs, in current Go trials, the mean amplitudes of the parietal P3 component between the 300‐ and 420‐ms time window were analyzed using a three‐way repeated‐measures ANOVA as a function of the dangerousness of the previous trial, the dangerousness of the current trial and the three SROIs. The peak amplitudes of the P2 component between the 160‐ and 240‐ms time window and the peak amplitudes of the N2 component between the 240‐ and 300‐ms time window, together with the mean amplitudes of the frontal P3 component between the 320‐ and 420‐ms time window, were analyzed using two‐way repeated‐measures ANOVAs as a function of the dangerousness of the previous trial and the dangerousness of the current trial. In contrast, in the NoGo trials, the mean amplitudes of the parietal P3 component between the 300‐ and 460‐ms time window were analyzed using a three‐way repeated‐measures ANOVA as a function of the dangerousness of the previous trial, the dangerousness of the current trial and the three SROIs. The peak amplitudes of the P2 component between the 160‐ and 240‐ms time window and the peak amplitudes of the N2 component between the 240‐ and 280‐ms time window, together with the mean amplitudes of the frontal P3 component between the 320‐ and 460‐ms time window, were analyzed using two‐way repeated‐measures ANOVAs as a function of the dangerousness of the previous trial and the dangerousness of the current trial. The degrees of freedom of the *F*‐ratio were corrected according to the Greenhouse‐Geisser method, and multiple comparisons were adjusted for with the Bonferroni method. The effect sizes are presented as partial eta squared values ($\eta_{p}^{2}$) for the ANOVAs and as Cohen's ds for the *t* tests. Statistical analyses were performed with SPSS (Version 23, http://scicrunch.org/resolver/SCR_002865).

## 3 RESULTS

### 3.1 Behavioral results

**Figure 4:**
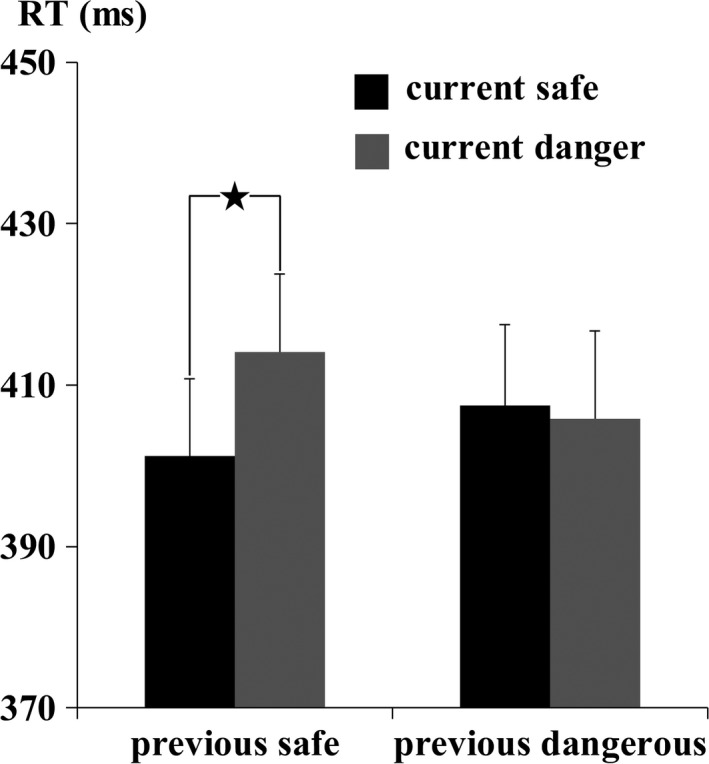
Results of the behavioral test. The figure presents the mean reaction times as a function of the dangerousness of the previous trial and the current trial in current Go trials. The error bars represent one standard error of the mean (*SE*)

**Figure 5:**
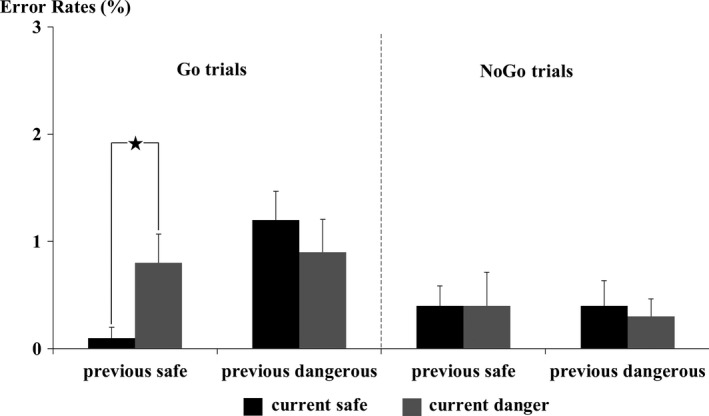
Results of the behavioral test. The figure presents the mean error rates as a function of the dangerousness of the previous trial and the current trial separately in current Go and NoGo trials. The error bars represent one standard error of the mean (*SE*)

The RTs were only analyzed in current Go trials (Figure 4). The results identified a significant main effect of the dangerousness of the current trial [*F* (1, 19) = 10.46, *p *=* *0.004, $\eta_{p}^{2}$ = 0.36] and a significant two‐way interaction between the dangerousness of the previous trial and the dangerousness of the current trial [*F* (1, 19) = 7.51, *p *=* *0.01, $\eta_{p}^{2}$ = 0.28]. Subsequent paired *t* tests indicated that the mean RTs for the sD condition (414 ± 43 ms) were longer than those for the sS condition (401 ± 43 ms; *t* (19) = 5.13, *p *<* *0.001, Cohen's *d* = 1.15). In contrast, the mean RTs for the dD condition (406 ± 49 ms) did not significantly differ from those for the dS condition (407 ± 43 ms; *t* (19) = 0.47, *p *=* *0.65, Cohen's *d* = 0.10). Regarding the error rates (Figure 5), analysis of current Go trials identified a significant main effect of the dangerousness of the current trial [*F* (1, 19) = 8.14, *p *=* *0.01, $\eta_{p}^{2}$ = 0.30] and a significant two‐way interaction between the dangerousness of the previous trial and the dangerousness of the current trial [*F* (1, 19) = 5.00, *p *=* *0.04, $\eta_{p}^{2}$ = 0.21]. Subsequent paired *t* tests indicated that the mean error rate for the sD condition (1.20 ± 1.20%) was larger than that for the sS condition (0.10 ± 0.45%; *t* (19) = 4.07, *p *=* *0.001, Cohen's *d* = 0.91). In contrast, the mean error rate for the dD condition (0.90 ± 1.37%) did not significantly differ from that for the dS condition (0.80 ± 1.20%; *t* (19) = 0.30, *p *=* *0.77, Cohen's *d* = 0.07]. In current NoGo trials, however, the results identified insignificant main effects and interaction (all *p*‐values >0.79) (Figure 5).

### 3.2 ERP results

**Figure 6:**
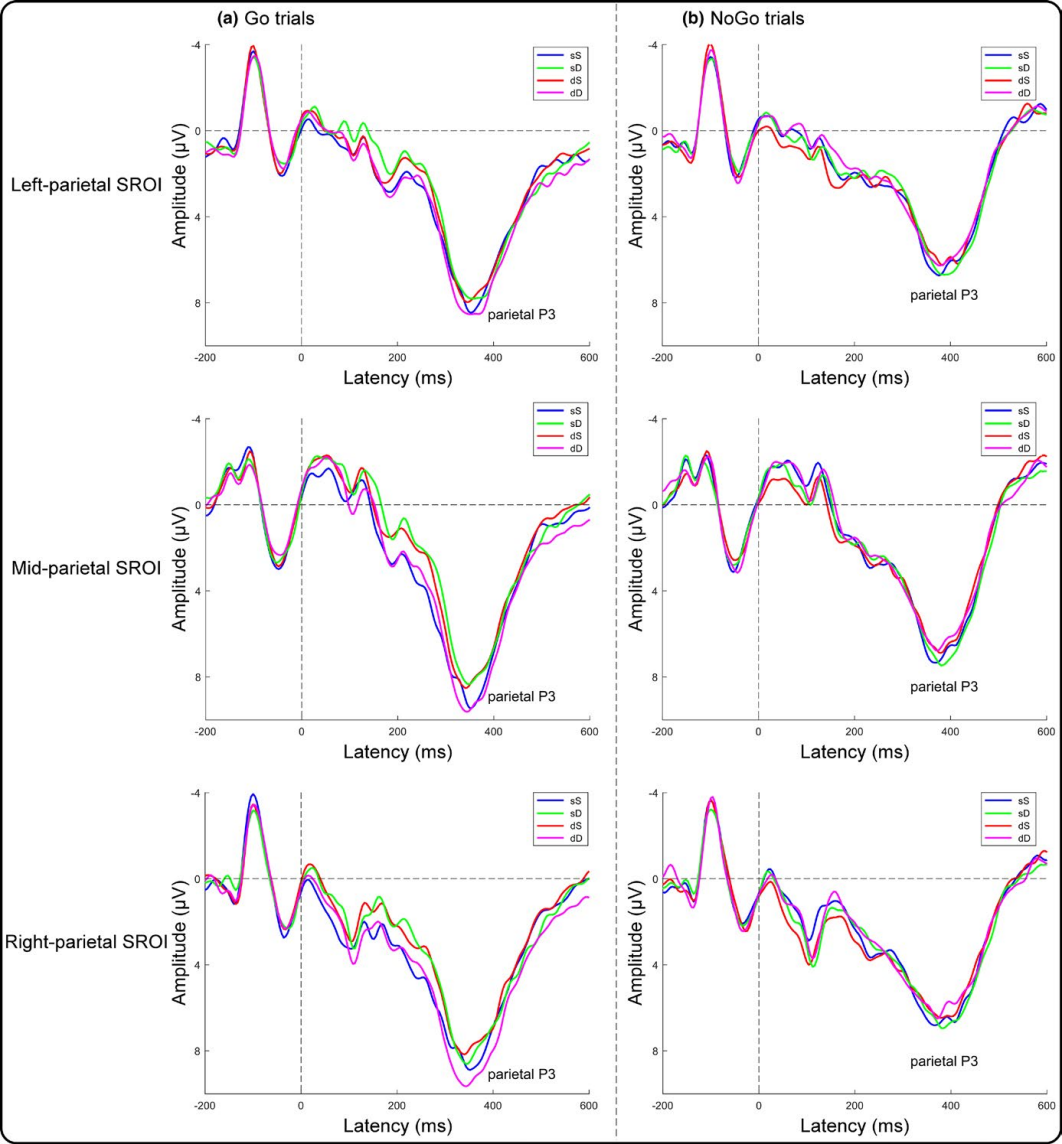
Target‐locked ERPs for the left (the mean of the CP3, CP5, P3, and P5 electrodes), middle (the mean of the CPz, P1, Pz, and P2 electrodes), and right (the mean of the CP4, CP6, P4, and P6 electrodes) parietal SROIs. (a) Group averages as a function of the dangerousness of the previous trial and the current trial in current Go trials at the three parietal SROIs. (b) Group averages as a function of the dangerousness of the previous trial and the current trial in current NoGo trials at the three parietal SROIs

In current Go trials (Figure 6), a three‐way repeated‐measures ANOVA of the parietal P3 amplitudes revealed significant two‐way interactions between the dangerousness of the previous trial and the dangerousness of the current trial and between the dangerousness of the current trial and the three SROIs. Moreover, a significant three‐way interaction was identified (Table 1). We then performed three separate two‐way repeated‐measures ANOVAs for the three SROIs. In the left parietal SROI, neither the main effects nor the interaction reached significance (all *p*‐values >0.21). In the middle parietal SROI, the main effects did not reach significance (all *p*‐values >0.22), but a significant two‐way interaction was identified [*F* (1, 19) = 6.30, *p *=* *0.02, $\eta_{p}^{2}$ = 0.25]. Subsequent paired *t* tests indicated that the parietal P3 amplitude was more positive for the dD condition (8.33 ± 4.78 μV) than for the dS condition (7.33 ± 4.24 μV; *t* (19) = 2.50, *p *=* *0.02, Cohen's *d* = 0.56). In contrast, the parietal P3 amplitude for the sD condition (7.16 ± 4.71 μV) did not significantly differ from that for the sS condition (7.94 ± 4.54 μV; *t* (19) = 1.48, *p *=* *0.16, Cohen's *d* = 0.33). In the right parietal SROI, a significant main effect of the dangerousness of the current trial [*F* (1, 19) = 4.94, *p *=* *0.04, $\eta_{p}^{2}$ = 0.21] and a significant two‐way interaction [*F* (1, 19) = 8.42, *p *=* *0.009, $\eta_{p}^{2}$ = 0.31] were identified. Subsequent paired *t* tests indicated that the parietal P3 amplitude was more positive for the dD condition (8.64 ± 4.77 μV) than for the dS condition (7.24 ± 4.40 μV; *t* (19) = 4.06, *p *=* *0.001, Cohen's *d* = 0.91). In contrast, the parietal P3 amplitude for the sD condition (7.44 ± 4.58 μV) did not significantly differ from that for the sS condition (7.76 ± 4.41 μV; *t* (19) = 0.77, *p *=* *0.45, Cohen's *d* = 0.17). Nevertheless, a three‐way repeated‐measures ANOVA of the parietal P3 amplitudes in current NoGo trials revealed no significant main effects or interactions (Table 1).

**Table 1:** ANOVA results (*F*‐values, *p*‐values, and partial eta squared values) of the mean amplitudes of the parietal P3 components as a function of the dangerousness of the previous trial, the dangerousness of the current trial, and the three SROIs separately for current Go and NoGo trials

Factors	*df*	Current Go trials			Current NoGo trials		
		*F*	*p*	$\eta_{p}^{2}$	*F*	*p*	$\eta_{p}^{2}$
Dangerousness of the previous trial	1, 19	2.36	0.14	0.11	3.66	0.07	0.16
Dangerousness of the current trial	1, 19	1.47	0.24	0.07	0.09	0.77	0.004
SROIs	2, 38	0.90	0.39	0.05	0.89	0.41	0.05
Dangerousness of the previous trial × Dangerousness of the current trial	1, 19	5.29	0.03*	0.22	0.001	0.97	0.000
Dangerousness of the previous trial × SROIs	2, 38	0.23	0.74	0.01	1.07	0.34	0.05
Dangerousness of the current trial × SROIs	2, 38	5.74	0.01**	0.23	0.32	0.66	0.02
Dangerousness of the previous trial × Dangerousness of the current trial × SROIs	2, 38	6.20	0.007**	0.25	0.15	0.84	0.008

*Note*

*df*, degrees of freedom.

*p ≤* *0.05, **p ≤* *0.01

**Figure 7:**
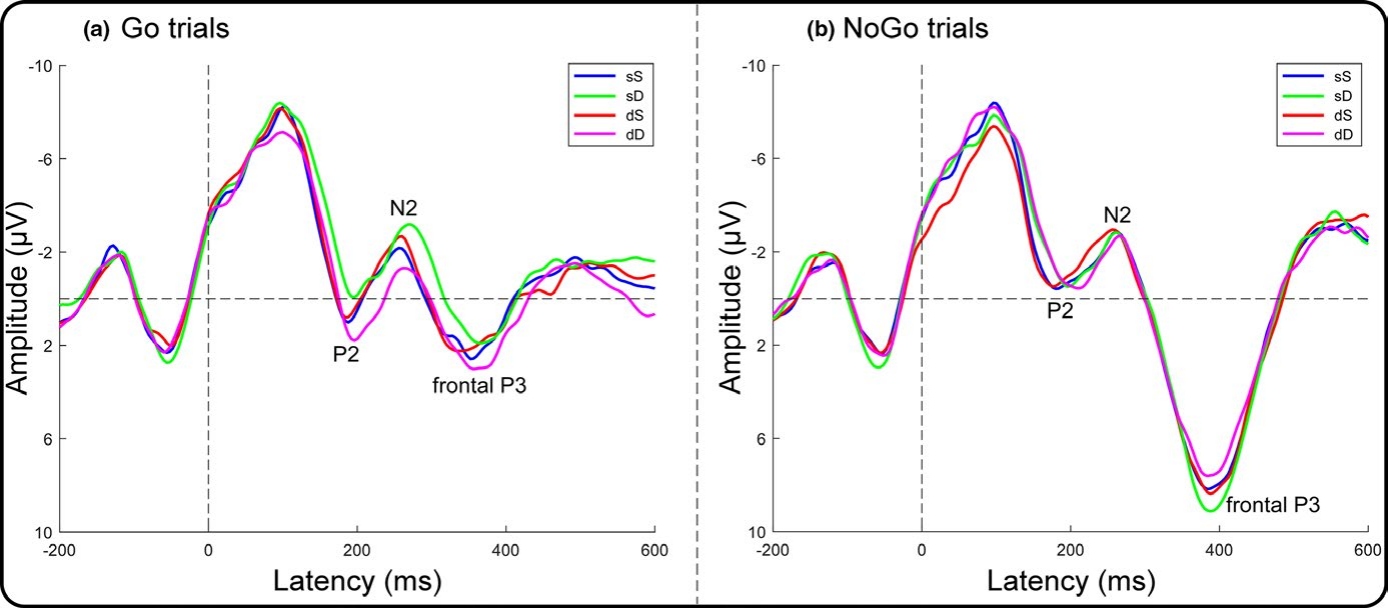
Target‐locked ERPs for the mid‐frontal area (the mean of the F1, Fz, F2, and FCz electrodes). (a) Group averages as a function of the dangerousness of the previous trial and the current trial in current Go trials. (b) Group averages as a function of the dangerousness of the previous trial and the current trial in current NoGo trials

In current Go trials (Figure 7), a two‐way repeated‐measures ANOVA of the P2 amplitudes revealed a significant main effect of the dangerousness of the previous trial together with a significant interaction between the dangerousness of the previous trial and the dangerousness of the current trial (Table 2). Subsequent paired *t* tests indicated that the P2 amplitude was more positive for the dD condition (3.00 ± 4.37 μV) than for the dS condition (1.91 ± 3.85 μV; *t* (19) = 2.14, *p *=* *0.05, Cohen's *d* = 0.48). In contrast, the P2 amplitude for the sD condition (1.03 ± 4.17 μV) did not significantly differ from that for the sS condition (2.10 ± 4.25 μV; *t* (19) = 1.67, *p *=* *0.11, Cohen's *d* = 0.37). Analysis of the N2 peak amplitudes and the frontal P3 mean amplitudes revealed that none of the main effects or interactions reached significance (Table 2). Furthermore, two‐way repeated‐measures ANOVAs of the P2, N2, and frontal P3 components in current NoGo trials revealed no significant main effects or interactions (Table 2).

**Table 2:** ANOVA results (*F*‐values, *p*‐values, and partial eta squared values) of the mean amplitudes of the P2, N2, and frontal P3 components as a function of the dangerousness of the previous trial, and the dangerousness of the current trial separately for current Go and NoGo trials

Component	Factors	*df*	Current Go trials			Current NoGo trials		
			*F*	*p*	$\eta_{p}^{2}$	*F*	*p*	$\eta_{p}^{2}$
P2	Dangerousness of the previous trial	1, 19	14.39	0.001**	0.43	0.12	0.73	0.006
	Dangerousness of the current trial	1, 19	0.00	0.99	0.00	0.03	0.86	0.002
	Dangerousness of the previous trial × Dangerousness of the current trial	1, 19	8.35	0.009**	0.31	0.49	0.49	0.03
N2	Dangerousness of the previous trial	1, 19	0.87	0.36	0.04	0.13	0.73	0.007
	Dangerousness of the current trial	1, 19	0.83	0.37	0.04	0.19	0.67	0.01
	Dangerousness of the previous trial × Dangerousness of the current trial	1, 19	3.88	0.06	0.17	0.37	0.55	0.02
Frontal P3	Dangerousness of the previous trial	1, 19	3.58	0.07	0.16	3.37	0.08	0.15
	Dangerousness of the current trial	1, 19	0.20	0.66	0.01	0.04	0.84	0.002
	Dangerousness of the previous trial × Dangerousness of the current trial	1, 19	1.83	0.19	0.09	0.97	0.34	0.05

*Note*

*df*, degrees of freedom.

**p ≤* *0.01

## 4 DISCUSSION

This study aimed to investigate whether processing a prepared response when facing a dangerous object influences the resolution of a subsequent dangerous trial. The following hypothesis was suggested: The attentional resources assigned to the dangerous target in the current trial would be affected (more attentional resources would be recruited) by the avoidance motivation elicited by the dangerous target in the previous trial. To test this hypothesis, this study used the experimental paradigm from Liu et al. (2017) and manipulated the dangerousness of the target objects in the previous trial (previous safe vs. previous dangerous), the dangerousness of the target objects in the current trial (current safe vs. current dangerous) and the Go/NoGo factor of the current trial (current Go vs. current NoGo). We hypothesized that in current Go trials, the motor interference effect obtained from the differences in the mean RTs and the error rates between current dangerous and current safe trials would be lower when these trials were preceded by a dangerous trial (dD minus dS) than when these trials were preceded by a safe trial (sD minus sS). In addition, the differences in attentional resources allocated to current dangerous trials minus current safe trials would be larger when these trials were preceded by a dangerous trial (dD minus dS) than when these trials were preceded by a safe trial (sD minus sS). Therefore, in current Go trials, reductions in the differences in the RTs and error rates should be observed between the dD and dS conditions compared with between the sD and sS conditions. Moreover, the differences in the P2 and parietal P3 amplitudes should be larger between the dD and dS conditions than between the sD and sS conditions. However, in current NoGo trials, attentional resources allocated to the targets were not influenced by target dangerousness. Therefore, we speculated that an insignificant effect as a function of the dangerousness of the current trial and the dangerousness of the previous trial would be observed in both behavioral and ERP results.

The behavioral results revealed a classical motor interference effect in the trials that were preceded by a safe trial, as evidenced by a longer RT and a larger error rate for the sD condition than for the sS condition in current Go trials. However, the motor interference effect was diminished in the trials that were preceded by a dangerous trial, as indicated by the lack of a significant difference in the mean RTs and error rates between the dD and dS conditions in current Go trials. The behavioral results suggested that the processing of a previous dangerous trial reduced the differences in the RTs and errors between subsequent dangerous and safe trials. At the neural level, analysis of the ERP data in current Go trials revealed a more positive parietal P3 amplitude in the dD condition than in the dS condition over the middle and right parietal regions. However, the parietal P3 amplitude of the sD condition did not significantly differ from that of the sS condition over the left, middle and right parietal regions. These results indicated an increase in the parietal P3 amplitude in dangerous versus safe trials that were preceded by a dangerous trial (dD relative to dS) relative to the parietal P3 amplitude in dangerous versus safe trials that were preceded by a safe trial (sD relative to sS). Because the parietal P3 component has been suggested to reflect cognitive resource assignment (Isreal et al., 1980), these results support the hypothesis that the difference in attentional resources allocated to current dangerous trials compared with current safe trials is larger when the current trial is preceded by a dangerous trial (dD minus dS) than when it is preceded by a safe trial (sD minus sS). The hypothesis was also supported by the P2 component results. Current dangerous trials elicited more positive P2 amplitudes than current safe trials when preceded by a dangerous trial in current Go trials. However, this P2 effect was diminished in trials that were preceded by a safe trial. The results indicated that the processing depth was deeper for dangerous targets than for safe targets when the previous trial was dangerous in early processing. In contrast, analyses of the error rates and the ERP components in current NoGo trials revealed that none of the main effects or interactions reached significance. The null effects in current NoGo trials probably emerged because attentional resources allocated to the targets were not influenced by the target dangerousness in the NoGo trials, which has been evidenced by Liu et al. (2017). Additionally, analysis of the N2 and frontal P3 amplitudes revealed insignificant main effects or interactions, which indicated that these two components were not associated with the hypothesis.

In summary, processing a prepared response when facing a dangerous object in a previous trial may influence the attentional resources allocated to a subsequent dangerous trial, thus facilitating RTs and reducing errors in consecutive dangerous trials. Specifically, the avoidance motivation elicited by a dangerous target in a preceding trial indicated a dangerous situation, and more attentional resources were prepared to evaluate the target in the subsequent trial. If the subsequent trial contained dangerous elements, deeper processing of the dangerous target may have recruited more attentional resources and therefore facilitated the responses to the dangerous target. However, processing of a current safe trial does not require the use of the previously recruited resources because these resources are not needed for a safe target. In contrast, a safe target in a previous trial indicates that nothing important occurred in the environment, and a classical motor interference effect emerged in the trials that were preceded by a safe trial. These results are consistent with the findings that the sequential trial effect can be modulated by avoidance motivation (Hengstler et al., 2014), and they present new evidence that phase (short‐term) avoidance motivation can also modulate the sequential trial effect based on the motor interference effect from dangerous objects. Generally, previous studies have found that the sequential trial effect can be modulated by emotional states (i.e., negative affect). In such studies, researchers induced a participant's affect prior to a conflict task (the Flanker or the Stroop tasks). Affect has been manipulated by multiple approaches, including the presentation of emotional music pieces (van Steenbergen, Band, & Hommel, 2010), emotional film clips (Schuch & Koch, 2015), success or failure feedback on an IQ test or tests measuring social perception skills (Schuch, Zweerings, Hirsch, & Koch, 2017). The study results identified an increase in the sequential trial effect in conditions of negative affect versus positive affect. The results suggested enhanced cognitive control in a negative emotional state compared with a positive emotional state. In this study, pictures of dangerous or threatening objects can be considered negative emotional stimuli because they can induce unpleasant or fearful affects. Moreover, the right‐side parietal asymmetry found in this study is consistent with the findings of Wright, He, Shapira, Goodman, and Liu (2004), who reported more extensive functional activity for mutilation pictures (which can elicit a more negative affect) than for threat pictures over the right posterior ventral cortex (Wright et al., 2004). Therefore, whether modulation of the sequential trial effect by avoidance motivation and modulation by negative affect share common mechanisms is an interesting question for future research.

Another interesting topic related to this study is whether the sequential trial effect based on the motor interference effect from dangerous objects can be modulated by the Go/NoGo factor of the previous trial. Although this study did not manipulate the Go/NoGo factor of the previous trial, this idea could be explored by performing a four‐way repeated‐measures ANOVA of the parietal P3 amplitudes as a function of the three parietal SROIs, the Go/NoGo factor of the current trial, the dangerousness of the previous trial, and the dangerousness of the current trial. The results indicated that the target dangerousness of the previous trial significantly interacted with the Go/NoGo factor of the current trial [*F* (1, 19) = 4.87, *p *=* *0.04, $\eta_{p}^{2}$ = 0.20]. Although the subsequent analysis revealed insignificant differences between a previous dangerous condition and a previous safe condition in both Go (7.69 ± 4.03 μV for a previous dangerous condition; 7.40 ± 3.96 μV for a previous safe condition; *t* (19) = 1.54, *p *=* *0.14) and NoGo (5.33 ± 3.09 μV for a previous dangerous condition; 5.67 ± 2.95 μV for a previous safe condition; *t* (19) = 1.91, *p *=* *0.07) trials, the significant two‐way interaction indicated that processing a dangerous or safe object in a previous trial influences the subsequent Go and NoGo trial processing differently. The trend of larger parietal P3 amplitude in the previous dangerous condition than in the previous safe condition in current Go trials might be because processing a dangerous object in a previous trial increases the attentional resources allocated to subsequent Go trials. Combined with previous findings that there is a conflict experience related to the dangerous objects only in Go trials (Liu et al., 2017), which might increase attentional resources allocated to subsequent trials. We further speculate that executing a prepared response toward a dangerous object (dangerous and Go condition) in a previous trial would increase the attentional resources recruited for subsequent Go trials. Thus, the sequential trial effect based on the motor interference effect from dangerous objects should increase when the previous trial and the current trial both correspond to the Go condition. This hypothesis could be tested via further manipulating the Go/NoGo factor of the previous trial based on the current design in future research.

## 5 CONCLUSION

This study aimed to investigate whether the processing of a prepared response when facing a dangerous object influences the resolution of a subsequent dangerous trial. The behavioral data indicated that in current Go trials, a classical motor interference effect from dangerous objects in trials preceded by a safe trial (i.e., longer RTs and greater error rates in the sD vs. sS conditions) was present. However, this motor interference effect diminished in trials that were preceded by a dangerous trial (i.e., insignificant differences in the RTs and error rates between the dD and dS conditions). The ERP results indicated that in current Go trials, increases in the P2 and parietal P3 amplitudes were observed in dangerous versus safe trials when these trials were preceded by a dangerous trial (i.e., the dD relative to dS conditions) compared with when dangerous versus safe trials were preceded by a safe trial (i.e., the sD relative to sS conditions). In summary, the results presented here support a possible mechanism for the sequential trial effect based on the motor interference effect from dangerous objects: Processing a dangerous target in a previous trial may induce an avoidance effect, which may indicate potentially dangerous situations and warrant a more careful examination of the current situation. Examination of the current situation leads to the effect of the P2 component in early processing. Furthermore, more attentional resources were recruited to process dangerous targets in trials that were preceded by a dangerous trial, which led to the effect of the parietal P3 component. The increased attentional resources subsequently led to accelerated RTs and reduced error rates in the consecutive dangerous condition. Obviously, the results indicate that the processing of a prepared response when facing a dangerous object in a previous trial may influence the resolution of a subsequent dangerous trial. However, the results from current NoGo trials revealed that none of the main effects or interactions corresponding to error rates or ERP components reached significance, indicating that the avoidance motivation elicited by a dangerous target in a previous trial may influence subsequent trial processing only if the prepared response needs to be executed.
